# Supplementary material for: Human Leukocyte Antigen-G is enriched in presence of trypanosome in the dermis of individuals exposed to gambiense Human African Trypanosomiasis in Guinea and Côte d’Ivoire
Source: PLoS Negl Trop Dis. 2026 Mar 9;20(3):e0014085. doi: 10.1371/journal.pntd.0014085 (PMC12987593; doi:10.1371/journal.pntd.0014085)
Supplement: S1 Table — CATTwb/ CATTp: card agglutination test for trypanosomiasis on whole blood/ plasma; TL: immune trypanolysis test; RDT: Rapid Diagnostic Test for HAT; mAECT BC/ LN aspirate: mini anion-exchange column technique on buffy coat/ lymph node aspirate; WBC: white blood cells; CSF: cerebrospinal fluid; *Highest plasma dilution with a positive result; S1 and S2: HAT stage 1 and 2. (DOCX) [file pntd.0014085.s003.docx]

| **Tests** | | **Serology** | | | **Parasitology** | | |
| --- | --- | --- | --- | --- | --- | --- | --- |
|  |  | **Screening** | **Validation** | **Trypanolysis** | **Confirmation** | **Staging** | |
| **Groups** | | CATTwb / RDT | CATTp* | TL | mAECT BC / LN aspirate observation | Parasites in CSF | WBC in CSF |
| **Control (CTR)** | | - | - | - / + | - |  | |
| **Seropositive**  **(SERO)** | **TL-** | + | ≧ 1/4 | - | - |  | |
|  | **TL+** |  |  | + |  |  |  |
| **Confirmed**  **cases** | **S1** | + | ≧ 1/4 | - /+ | + | no | 0-5 |
|  | **S2** |  |  |  |  | yes | >5 |

### **Table S1. Diagnostic tests and group definition.**

CATTwb / CATTp: card agglutination test for trypanosomiasis on whole blood / plasma; TL: immune trypanolysis test; RDT: Rapid Diagnostic Test for HAT; mAECT BC / LN aspirate: mini anion-exchange column technique on buffy coat / lymph node aspirate; WBC: white blood cells; CSF: cerebrospinal fluid; *Highest plasma dilution with a positive result; S1 and S2: HAT stage 1 and 2.
