# Supplementary material for: Human Leukocyte Antigen-G is enriched in presence of trypanosome in the dermis of individuals exposed to gambiense Human African Trypanosomiasis in Guinea and Côte d’Ivoire
Source: PLoS Negl Trop Dis. 2026 Mar 9;20(3):e0014085. doi: 10.1371/journal.pntd.0014085 (PMC12987593; doi:10.1371/journal.pntd.0014085)
Supplement: S2 Table — A) at enrolment and B) during follow-up, and between result of trypanolysis test and parasitological status in the dermis C) at enrolment and D) during follow-up. a Logistic regression and b logistic mixed regression were applied to investigate the association between explanatory variables (IHC, HAT focus, age, sex, fever, pruritus and dermatitis) and parasitology (A and B) or trypanolysis (C and D) status in the blood. Significant results at P < 0.05. mAECT BC/ LN aspirate: mini anion-exchange column technique on buffy coat/ lymph node aspirate; IHC: immuno-histochemistry. (DOCX) [file pntd.0014085.s004.docx]

| **A** |  |  | **Adjusted B^(a)^** | **Std Error** | **P-value** |
| --- | --- | --- | --- | --- | --- |
|  | **mAECT** (0 vs 1) | |  |  |  |
|  |  |  |  |  |  |
|  |  | **IHC (anti-ISG65)** | -0.73 | 0.64 | 0.26 |
|  |  |  |  |  |  |
| **B** |  |  | **Adjusted B^(b)^** | **Std Error** | **P-value** |
|  | **mAECT** (0 vs 1) | |  |  |  |
|  |  |  |  |  |  |
|  |  | **IHC (anti-ISG65)** | -0.56 | 0.53 | 0.30 |

| **C** |  | |  | | | **Adjusted B^(a)^** | **Std Error** | | **P-value** |
| --- | --- | --- | --- | --- | --- | --- | --- | --- | --- |
|  | **Trypanolysis test** (0 vs 1) | | |  | | |  | |  |
|  |  | |  |  | | |  | |  |
|  |  | **IHC (anti-ISG65)** | | 1.67 | | | 0.60 | | 0.99 |
|  |  |  | |  | | |  | |  |
| **D** |  |  | | | | **Adjusted B^(b)^** | **Std Error** | | **P-value** |
|  | **Trypanolysis test** (0 vs 1) | | | |  | |  | |  |
|  |  |  | | |  | |  | |  |
|  |  | **IHC (anti-ISG65)** | | | -0.38 | | 0.72 | | 0.60 |
|  |  | |  | |  | | |  |  |

### **Table S2. Associations between parasitological status in the blood and in the dermis A) at enrolment and B) during follow-up, and between result of trypanolysis test and parasitological status in the dermis C) at enrolment and D) during follow-up.**

^a^ Logistic regression and ^b^ logistic mixed regression were applied to investigate the association between explanatory variables (IHC, HAT focus, age, sex, fever, pruritus and dermatitis) and parasitology (A and B) or trypanolysis (C and D) status in the blood. Significant results at P<0.05. mAECT BC / LN aspirate: mini anion-exchange column technique on buffy coat / lymph node aspirate; IHC: immuno-histochemistry.
