# Supplementary material for: Human Leukocyte Antigen-G is enriched in presence of trypanosome in the dermis of individuals exposed to gambiense Human African Trypanosomiasis in Guinea and Côte d’Ivoire
Source: PLoS Negl Trop Dis. 2026 Mar 9;20(3):e0014085. doi: 10.1371/journal.pntd.0014085 (PMC12987593; doi:10.1371/journal.pntd.0014085)
Supplement: S3 Table — A) at enrolment. B) during follow-up. sHLA-G, soluble human leucocyte antigen-G. a Linear regression and b linear mixed regression were applied to investigate the association between explanatory variables parasitological status in the blood (mAECT) and sHLA-G plasmatic level adjusted on HAT focus, age, sex, fever, pruritus and dermatitis status. Significant results at P < 0.05. (DOCX) [file pntd.0014085.s005.docx]

|  |  |  |  |  |  |
| --- | --- | --- | --- | --- | --- |
| **A** |  |  | **Adjusted B^(a)^** | **Std Error** | **P-value** |
|  | **sHLA-G level (log+1)** | |  |  |  |
|  |  |  |  |  |  |
|  | **mAECT** | | **0.75** | 0.16 | **1.18.10^-5^** |
|  |  |  |  |  |  |
|  |  |  |  |  |  |
|  |  |  |  |  |  |
|  |  |  | **Adjusted B^(b)^** | **Std Error** | **P-value** |
| **B** | **sHLA-G level (log+1)** | |  |  |  |
|  |  |  |  |  |  |
|  | **mAECT** | | **0.44** | 0.16 | **2.86.10^-3^** |
|  |  |  |  |  |  |
|  |  |  |  |  |  |

### **Table S3. Associations between sHLA-G plasmatic level and parasitological status in the blood.**

A) at enrolment. B) during follow-up. sHLA-G, soluble human leucocyte antigen-G. ^a^ Linear regression and ^b^ linear mixed regression were applied to investigate the association between explanatory variables parasitological status in the blood (mAECT) and sHLA-G plasmatic level adjusted on HAT focus, age, sex, fever, pruritus and dermatitis status. Significant results at P<0.05.
