# Supplementary material for: Human Leukocyte Antigen-G is enriched in presence of trypanosome in the dermis of individuals exposed to gambiense Human African Trypanosomiasis in Guinea and Côte d’Ivoire
Source: PLoS Negl Trop Dis. 2026 Mar 9;20(3):e0014085. doi: 10.1371/journal.pntd.0014085 (PMC12987593; doi:10.1371/journal.pntd.0014085)
Supplement: S4 Table — A) at enrolment. B) during follow-up. sHLA-G, soluble human leucocyte antigen-G. a Linear regression and b linear mixed regression were applied to investigate the association between explanatory variables trypanolysis test result and dermatitis adjusted on HAT focus, age, sex, fever, pruritus and dermatitis status. Significant results at P < 0.05. (DOCX) [file pntd.0014085.s006.docx]

| **A** |  |  | **Adjusted B^(a)^** | **Std Error** | **P-value** |
| --- | --- | --- | --- | --- | --- |
|  | **sHLA-G level (log+1)** | |  |  |  |
|  |  |  |  |  |  |
|  | **Trypanolysis test** | | **0.52** | 0.13 | **9.86.10^-5^** |
|  |  |  |  |  |  |
|  |  |  |  |  |  |
|  |  |  |  |  |  |
|  |  |  | **Adjusted B^(b)^** | **Std Error** | **P-value** |
| **B** | **sHLA-G level (log+1)** | |  |  |  |
|  |  |  |  |  |  |
|  | **Trypanolysis test** | | **0.37** | 0.12 | **2.25.10^-3^** |
|  |  |  |  |  |  |
|  |  |  |  |  |  |

### **Table S4. Associations between sHLA-G plasmatic level and results of trypanolysis tests.**

A) at enrolment. B) during follow-up. sHLA-G, soluble human leucocyte antigen-G. ^a^ Linear regression and ^b^ linear mixed regression were applied to investigate the association between explanatory variables trypanolysis test result and dermatitis adjusted on HAT focus, age, sex, fever, pruritus and dermatitis status. Significant results at P<0.05.
