# Supplementary material for: Human Leukocyte Antigen-G is enriched in presence of trypanosome in the dermis of individuals exposed to gambiense Human African Trypanosomiasis in Guinea and Côte d’Ivoire
Source: PLoS Negl Trop Dis. 2026 Mar 9;20(3):e0014085. doi: 10.1371/journal.pntd.0014085 (PMC12987593; doi:10.1371/journal.pntd.0014085)
Supplement: Attachment — Submitted filename: Review_HAT e HLA_G.docx [file pntd.0014085.s008.docx]

**Reviewer Comments**

**General Assessment**

This manuscript addresses an important and timely topic by investigating the differential expression of HLA-G in the skin of HAT-seropositive and HAT-seronegative individuals in Guinea and Côte d’Ivoire, and by comparing these findings to systemic sHLA-G levels. The study has the potential to contribute valuable insights into the immunopathology of HAT. Several methodological and interpretative issues must be addressed to strengthen the manuscript’s scientific rigor and clarity.

**Major Comments**

1. **Study Design and Control Group Definition**
   - The inclusion of individuals with a history of past HAT infection or previous seropositivity as “seronegative controls” in Côte d’Ivoire raises concerns. These individuals may have residual immune activation that could alter HLA-G expression, particularly sHLA-G, and therefore may not serve as appropriate negative controls.
   - **Recommendation**: Reassess the composition of the control group. Ideally, controls should be free of prior exposure or immune activation related to HAT.
2. **Flowchart and Case Definitions**
   - The manuscript would greatly benefit from a **flow diagram** illustrating the participant screening process, case definitions, inclusion/exclusion criteria, and final numbers per group.
   - **Recommendation**: Include a flowchart and specify the number of individuals in each group at each step.
3. **Clinical Characterization of Cases**
   - The manuscript lacks information on the **clinical presentation** of confirmed HAT cases.
   - **Recommendation**: Describe the clinical symptoms observed in HAT-positive participants.
4. **Use of Trypanolysis Test**
   - Trypanolysis is mentioned as part of the case definition, but is not consistently described with other diagnostic tests.
   - **Recommendation**: Provide a complete and consistent list of all diagnostic tests used in case definitions, preferably in a supplementary table.
5. **Follow-up Period**
   - The rationale and duration of follow-up sampling are not clearly stated.
   - **Recommendation**: Clarify the follow-up protocol and explain the choice of timepoints for sampling.
6. **Inclusion of Parasite-Positive Dermal Samples in Controls**
   - The inclusion of parasite-positive dermal samples among seronegative controls is problematic and not sufficiently justified.
   - **Recommendation**: Consider excluding these samples from the control group or provide a stronger rationale and discuss potential biases.

**Specific Comments**

- **Line 202**: Clarify whether all participants underwent the epidemiological interview or only confirmed cases. Also, specify the type of data that was collected.
- **Line 303**: Move demographic data to the Methods section to improve flow and clarity.
- **Lines 314–317**: The findings regarding the absence of association between blood and dermal parasites, as well as trypanolysis results, are not clearly aligned with the study's main objective and are not supported by referenced tables.
- **Line 343–344 / Table 3**: Explain why samples were collected at three different timepoints and make this more straightforward in the table legend.
- **Line 360 / Table 1**: Numbers in the text do not match those in Table 1. Please review and ensure consistency.

**Tables**

- **Table 1**: Reformat the presentation to align with how frequency data are reported. The current layout is confusing.
- **Tables 2 and 4**: Rearrange tables to display comparisons between cases and controls more clearly (consider inverting the order of groups for easier visual comparison).

**Conclusion**

The study provides promising data regarding the role of HLA-G in HAT. However, essential revisions are needed to improve methodological clarity, data presentation, and control group selection. Once these issues are addressed, the manuscript will be better positioned to make a significant contribution to the field.
