# Supplementary material for: Human Leukocyte Antigen-G is enriched in presence of trypanosome in the dermis of individuals exposed to gambiense Human African Trypanosomiasis in Guinea and Côte d’Ivoire
Source: PLoS Negl Trop Dis. 2026 Mar 9;20(3):e0014085. doi: 10.1371/journal.pntd.0014085 (PMC12987593; doi:10.1371/journal.pntd.0014085)
Supplement: Attachment — Submitted filename: Responses V3.docx [file pntd.0014085.s009.docx]

Dear editors, dear colleagues, dear Jayne,

We are happy to read that all reviewers appreciated our manuscript. We thank them for their careful reading and their precise and useful suggestions. We have addressed all their comments in the point-by-point response letter hereafter. The corresponding modifications are highlighted in the track-changes manuscript.

We acknowledge that the definition of the pictures inserted along the main text was poor, however, high-definition figures were uploaded at initial submission. We also understand that the new reference (Ref #15), corresponding to an article that was still in press at initial submission (N’Djetchi, PLoS NTDs, in press), would have been helpful as it seems to answer several of the reviewers’ comments.

Best regards,

Brice

**Comments to the Authors:**

**Reviewer #1:**

The methods are very clearly described - the authors have done a very good job of describing the technical details of their wet-lab experiments as well as their patient samples. The N numbers in each group are a key strength of the manuscript, given that virtually 100% of the data and interpretations are based on statistics. The regression models chosen for each analysis are good choices as far as I can tell.

Thank you.

**Reviewer #2:**

General Assessment

This manuscript addresses an important and timely topic by investigating the differential expression of HLA-G in the skin of HAT-seropositive and HAT-seronegative individuals in Guinea and Côte d’Ivoire, and by comparing these findings to systemic sHLA-G levels. The study has the potential to contribute valuable insights into the immunopathology of HAT. Several methodological and interpretative issues must be addressed to strengthen the manuscript’s scientific rigor and clarity.

Thank you. We have addressed all reviewer 2 comments hereafter.

We acknowledge that the definition of the pictures inserted along the main text was poor, however, high-definition figures were uploaded at initial submission and should have been available. We also understand that the new reference (Ref #15), corresponding to an article that was still in press at initial submission (N’Djetchi, PLoS NTDs, in press), would have been helpful as it seems to answer several of the reviewers’ comments.

Major Comments

1. **Study Design and Control Group Definition**
   - The inclusion of individuals with a history of past HAT infection or previous seropositivity as “seronegative controls” in Côte d’Ivoire raises concerns. These individuals may have residual immune activation that could alter HLA-G expression, particularly sHLA-G, and therefore may not serve as appropriate negative controls.
   - **Recommendation**: Reassess the composition of the control group. Ideally, controls should be free of prior exposure or immune activation related to HAT.

More details about subjects from Côte d’Ivoire are available in (Ref #15). Here, seronegative controls (CTR) included individuals who were all negative in CATTwb and / or RDT, and all negative in CATTp. CATT titration on diluted serum or plasma is useful for making therapeutical decisions (Simarro et al. TMIH 2002), yet CATT is not a test of cure as positivity in CATT can persist for month and up to two years after treatment (Lejon et al. PLoS NTD 2010). Here, one of the inclusion criteria was therefore negativity in CATTwb at enrolment, which we assumed to be a reasonable marker of the absence of an ongoing and/or recent infection. Moreover, among subjects in the CTR group, preliminary univariate analyses showed no difference in sHLA-G levels or dermal HLA-G profiles between naïve subjects and subjects with past HAT history. In addition, the data from individuals enrolled in the SERO or HAT groups, and who became negative in CATTwb at follow-up 1 and / or 2, were not included in the CTR group for follow-up analyses. Group assignment was only based on diagnostic results at baseline. Comments on the homogeneity of the CTR group were added in the Results section.

2. **Flowchart and Case Definitions**
   - The manuscript would greatly benefit from a **flow diagram** illustrating the participant screening process, case definitions, inclusion/exclusion criteria, and final numbers per group.
   - **Recommendation**: Include a flowchart and specify the number of individuals in each group at each step.

Two flowcharts were added in a new Fig S2.

3. **Clinical Characterization of Cases**
   - The manuscript lacks information on the **clinical presentation** of confirmed HAT cases.
   - **Recommendation**: Describe the clinical symptoms observed in HAT-positive participants.

As expected, the clinical symptoms observed in HAT-positive participants were not specific and the main clinical features are already detailed in references #13-15. A comment is now provided in the ‘Population and demographics’ sub-section of the Results: ‘Most clinical symptoms observed in confirmed HAT cases were not specific and their main clinical features are detailed in [1-3]. The two most frequent symptoms were the occurrence of swollen cervical lymph nodes and dermatological signs (dermatitis and / or pruritus).’

4. **Use of Trypanolysis Test**
   - Trypanolysis is mentioned as part of the case definition, but is not consistently described with other diagnostic tests.
   - **Recommendation**: Provide a complete and consistent list of all diagnostic tests used in case definitions, preferably in a supplementary table.

A new Table S1 now summarizes the diagnostic tests defining each group.

5. **Follow-up Period**
   - The rationale and duration of follow-up sampling are not clearly stated.
   - **Recommendation**: Clarify the follow-up protocol and explain the choice of timepoints for sampling.

As stated in the Methods section, Study enrolment sub-section, ‘The study was conducted in two study sites in Guinea (Boffa and Forecariah) and two in Côte d’Ivoire (Bonon and Sinfra) between December 2018 and April 2022. As described in detail in [14] and [15], all subjects were enrolled during medical surveys performed by the HAT National Control Programme in Guinea and the HAT National Elimination Programme in Côte d’Ivoire, according to WHO recommendations.’ Here, the follow-up visits were not strictly planed at the beginning of the study but rather organized according to the activities of the National Control Programmes. An additional comment is now provided.

6. **Inclusion of Parasite-Positive Dermal Samples in Controls**
   - The inclusion of parasite-positive dermal samples among seronegative controls is problematic and not sufficiently justified.
   - **Recommendation**: Consider excluding these samples from the control group or provide a stronger rationale and discuss potential biases.

A new Table S1 now summarizes the diagnostic tests defining each group. These reference tests are performed directly in the field (except for TL that is performed in a reference lab within a month) and recognized as the gold standards for defining a subject status in most international studies, including in references #14-15. The occurrence of extravascular dermal parasites is extemporaneously assessed in a research lab and is officially not considered for HAT diagnosis and treatment. Here, the group definition was standardized on recognized criteria to allow for comparisons. As commented in references #14-15, we were also surprised to identify parasite-positive dermal samples among some rare seronegative controls, which is currently a question that we are investigating.

Specific Comments

- **Line 202**: Clarify whether all participants underwent the epidemiological interview or only confirmed cases. Also, specify the type of data that was collected.

As stated, ‘each participant’ underwent an epidemiological interview. The type of data collected is described in [1-3] but these epidemiological data were not considered in the present study. As mentioned here, only ‘The following data were collected: age and sex.’ This was clarified.

- **Line 303**: Move demographic data to the Methods section to improve flow and clarity.

We think that these data are more related to the Results section.

- **Lines 314–317**: The findings regarding the absence of association between blood and dermal parasites, as well as trypanolysis results, are not clearly aligned with the study's main objective and are not supported by referenced tables.

Supporting data are now provided in a new Table S2.

- **Line 343–344 / Table 3**: Explain why samples were collected at three different timepoints and make this more straightforward in the table legend.

This is now clarified in the Table 3 legend.

- **Line 360 / Table 1**: Numbers in the text do not match those in Table 1. Please review and ensure consistency.

This was checked and no error was detected. The term ‘all time points’ in the text indicates the entire data set, but Table 1 only shows enrolment data.

Tables

- **Table 1**: Reformat the presentation to align with how frequency data are reported. The current layout is confusing.
- **Tables 2 and 4**: Rearrange tables to display comparisons between cases and controls more clearly (consider inverting the order of groups for easier visual comparison).

This was modified as much as possible.

Conclusion

The study provides promising data regarding the role of HLA-G in HAT. However, essential revisions are needed to improve methodological clarity, data presentation, and control group selection. Once these issues are addressed, the manuscript will be better positioned to make a significant contribution to the field.

We are happy to read that reviewer 2 appreciated our manuscript. We thank her / him for these precise and useful suggestions that we have all addressed. We understand that the new reference (Ref #15), corresponding to an article that was still in press at initial submission (N’Djetchi, PLoS NTDs, in press), would have been helpful as it seems to answer several of the reviewer’s comments.

**Reviewer #3:**

The objectives of the study are clearly articulated, with a clear testable hypothesis.
The study design could improve, particularly the figures including images, as they are being included in support of the main hypothesis.

The population is clearly described and is appropriate for the hypothesis being tested.
The sample size is sufficient, and the statistical analysis is valid. Nevertheless, there are important elements of figures 1 and S1 missing - and the information included in the layout should improve too.

We acknowledge that the definition of the pictures inserted along the main text was poor, however, high-definition figures were uploaded at initial submission. We have now provided each figure as an independent high-definition file.

I did not find anything concerning regarding ethical or regulatory elements.

**Results**

**Reviewer #1:**

The manuscript would benefit from higher quality images as well as a higher number of images. This is a significant issue with the manuscript. The image quality in figure 1 is poor to the point where even the computer-drawn arrows look pixelated. Presumably this was a file size issue? But in any case, the reader is left wondering how many of these IHC signals are actually real, which is particularly problematic in the context of the "diffuse halos" which are really weak signals (to the authors' credit - they point this out themselves and address it in the discussion). My suggestion would be to 1) improve the figure quality / sharpness and 2) add in several more positive staining samples to the supplemental figures so that the reader can see for themselves, rather than rely only on the numbers in the tables. And speaking to the tables - it would be much much nicer to present the key linear regressions as actual charts for the reader to evaluate. Only publishing the B and P values for all of these data is less satisfying for the reader.

We acknowledge that the definition of the pictures inserted along the main text was poor, however, high-definition figures were uploaded at initial submission. We have now provided each figure as an independent high-definition file.

**Reviewer #3:**

The analysis presented in the tables is thorough and matches the analysis plan.
Regarding the results, I have concerns with current Figure 1 and Figure S1. The authors address the issue of poor signal. I would suggest doing image processing - for instance, CLAHE (a plugin for Fiji) would help. At present, the point they aim to make is not very clear. The design of these figures could also improve as follows:

We acknowledge that the definition of the pictures inserted along the main text was poor, however, high-definition figures were uploaded at initial submission. We have now provided each figure as an independent high-definition file.

a) There should be representation of more than one sample and this should be clearly shown in the figure.

Yes, it is the case. 5 distinct samples are shown in Figure 1.

b) Currently, it is unclear whether the larger magnification/zoom-in come from the low magnification image shown, or not, if they are separate (which ideally, they should not be). The area of the zoom in should be clearly shown.

Figure 1 presents 5 distinct samples. This was clarified in the figure legend.

c) Scale bars are missing. They must be included in order to help the reader better understand the images.

Scale bars were added in all pictures.

d) Improving the image quality through pre-processing might allow a more quantitative approach.

We have initially envisaged a possible automatized quantification of the halo areas and / or intensity, but the *in-silico* ROI and target definitions were approximative and poorly reproducible, even after applying different thresholding methods. Hence, we opted for the more robust, yet tedious, manual quantification by comparing successive stained Vs. unstained sections.

e) Although the authors mention that the study is semi-quantitative regarding the images, and although the tables show these quantifications, it would be useful to include even the semi-quantitative graphs together with the figure - it helps the reader evaluate each figure standalone, as well as in the context of the tables and the general paper.

All raw numbers of delta HLA-G halos and delta HLA-G granular spots are provided in the new data source file Sup File 1. We think that the data syntheses presented in Tables 1 and 3 are too complex for visual representation in semi-quantitative graphs. Instead, we believe that having all values clearly visible in the same table allows a more subtle understanding and integration of the data set.

f) Where/if arrows are used, for the inserts, the arrows in the low magnification figure should be numbered. That number would correspond to the high-mag/zoom-in image. Right now it lacks structure.
Figure 1 is showing representative pictures from 5 distinct samples. This is now explained in the legend and more visible scale bars were added. Here, circles and arrows are only indicating the typical HLA-G localization as diffuse halos and granular brown spots, respectively, and on high magnification pictures only. Although HLA-G antigen quantification was performed on entire skin sections, HLA-G signal is not visible at larger scale.

g) The authors mention that it was not possible to have co-detection of HLA-G and parasite markers. It is not clear in the text why this is the case.

In our hands, the Bond Polymer Leica Kits necessary to amplify the signal up to visible levels for both ISG565 / CRD and HLA-G antigens detection, were not compatible for dual staining in optimal conditions. We fully agree it would have been ideal.

h) If a fully quantitative approach is possible for the images, clear methodology should be included as to how the analysis was performed.

As mentioned just before, we have initially envisaged a possible automatized quantification of the halo areas and / or intensity, but the *in-silico* ROI and target definitions were approximative and poorly reproducible, even after applying different thresholding methods. Hence, we opted for the more robust, yet tedious, manual quantification by comparing successive stained Vs. unstained sections. This was clearly explained in Methods section: ‘Semi-quantitative assessment of HLA-G loads in skin sections was remotely assessed by two independent biologists blind to group assignment and experimental procedures. Different parameters were first quality-checked to validate the exploitability of each slide. The readability of the slide was assessed by the integrity of the overall structure of the section. Then, we checked that the image definition was compatible with HLA-G detection. Non-readable images (blurry, out of focus, over / under exposed) were re-scanned once. Then, the presence of a minimal amount of dermis was also checked by controlling the thickness of the section (excluding the epidermis and an inward thickness equivalent to an epidermis to avoid any source of melanocytic contamination). Non-validated slides were not further considered for HLA-G detection. In quality-check validated slides, the presence of HLA-G was detected by observing granular brown spots and / or diffuse halos at x 40 magnification (Figure 1). The numbers of granular brown spots and diffuse halos per slide were quantified using the ZEN 3.9 software (Carl Zeiss) and deltas were calculated between two successive anti-HLA-G labelled slide and unstained negative control slide for each individual skin sample. In total, a validated slide was considered positive for the presence of HLA-G when the delta of the sum of diffuse halos and / or the delta of the sum of granular brown spots were equal or greater than 1. This allowed us to the assess the presence of soluble and / or membrane HLA-G in the dermal region of most samples.’

Another key point is that the raw data (for both ELISAs and imaging) that were used to derive the conclusions of the paper, should be included.

Yes, of course, we omitted this at initial submission. All raw data are now provided in the new data source file Sup File 1.

**Conclusions**

**Reviewer**

#1: There are three main conclusions in this paper.

1) the authors observe supportive data for a pre-existing conclusion that serum-HLAg levels coorelate with trypanosome infection. The correlations presented in this manuscript in this context are strong and indeed it appears that serum-HLAg could be considered a good biomarker in the Tbg infection context (though not a Tbg-specific biomarker - as the authors admit). I would prefer that the data be shown as individual data points rather than box-whiskers though. Easier for the reader to determine how strong it really is. Box-whisker for me is only appropriate when you have really very high N numbers.

The plots shown in Fig 2 and 3 are based on ELISA data detailed in Tables 1 and 3. Hence, both visual representations and precise numbers are available to the reader for a better understanding of the data set.

2) the authors observe that the "granular brown spots" of HLAg in the dermis correlate with the presence of trypanosomes. The correlation here is again strong. The expectation is that these HLAg signals are from cell-bound / transmembrane HLAg on some skin-infiltrating immune cells, which is also observed in other skin conditions.

The authors state that they were unable to investigate the cells expressing this HLAg. IHC does have its limitations and I do not think additional experiments are necessary for publication here; but I would say that the novelty of this finding would be significantly more impactful if the identity of these cells was determined. Presumably the extensive trypanosome literature could be consulted, identifying a small handful of candidate cell types that could be stained for in follow up studies. But indeed, these would need to be immunofluorescence studies instead of IHC (for colocalization). If the authors are capable / equipped for such studies, then the manucsript would be much improved.

Yes, we appreciate the reviewer’s comments. We are currently working more in depth on the immune response in the human skin in the context of another study that will require much more work, and we think this could likely clarify the question of the exact nature of these HLA-G+ cells.

Would the authors hypothesize that the HLAg expressing cells are playing some immunomodulatory role that helps the trypanosome infection remain "dormant" in the skin? Or would they hypothesize that this HLAg is directly trypanosome-suppressive? The authors touch on these topics in the discussion (lines 445-466) but I'm not sure what their hypotheses would be.

We indeed proposed that the HLA-G up-regulation observed at the surface of immune cells in presence of trypanosome infection in the dermis may be a way to modulate the immune surveillance level, or part of an internal regulatory system to control excessive inflammation. At this stage, it is difficult to elaborate much more on these aspects as further characterization of the immune response are required.

3) the authors observe that the "diffuse halos" of HLAg in the dermis correlate with the serum HLAg levels. This correlation is much much weaker than those above as far as I can tell from the P values. And given that the signal intensities from the "diffuse halos" are so weak, I'm hesitant to trust this correlation. From a logical perspective, a higher amount of circulating HLAg could very well facilitate the "leakage" of HLAg into the dermis so long as there is some mechanism for its transport out of the blood vessels. Is that where it is "coming from"? Or is the hypothesis that it is locally produced and secreted within the dermis? ... Nevertheless, I think the data supporting conclusion 3 are weak, but not so weak to prevent publication. I do however think that publishing a lot more of the raw data (many more of the IHC images) in the supplementals so that the reader can make their own interpretations is key.

By definition, it is true that the signals defined as diffuse halos were weak. The fact that visual detection of soluble HLA-G by IHC is possible may actually reflects rather high local concentrations of HLA-G molecules in the dermal matrix. Their origin remains however uncertain as these halos were not necessarily localized neither at the vicinity of vascular structures nor around brown spots.

All raw data, including numbers of delta HLA-G halos and delta HLA-G granular spots, are now provided in the new data source file Sup File 1. We acknowledge that the definition of the pictures inserted along the main text was poor, however, high-definition figures were uploaded at initial submission. We have now provided each figure as an independent high-definition file.

**Reviewer #3:**

The conclusions are supported by the data, but better images will help make the point. The limitations of the analysis are well described. The public health relevance is addressed, and the authors discuss how the data can help us advance our understanding of the topic. As mentioned above though, the figures including images are not 100% convincing - by both B&C and a relative lack of clarity in the annotations shown.

Thank you. We acknowledge that the definition of the pictures inserted along the main text was poor, however, high-definition figures were uploaded at initial submission. We have now provided each figure as an independent high-definition file.

**Editorial and Data Presentation Modifications?**

**Reviewer #1:**

Improved figure quality as discussed. Typos throughout that must be fixed.

Yes, as previously stated, we acknowledge that the definition of the pictures inserted along the main text was poor, however, high-definition figures were uploaded at initial submission. We have now provided each figure as an independent high-definition file.

**Reviewer #3:**

As suggested above - Figures 1 and S1 can be improved in quality, annotation and content.
The raw data of both ELISAs and images, should be included.

Yes, as previously stated, all raw data are now provided in the new data source file Sup File 1. We acknowledge that the definition of the pictures inserted along the main text was poor, however, high-definition figures were uploaded at initial submission. We have now provided each figure as an independent high-definition file.

**Summary and General Comments**

**Reviewer #1:**

Working with patient-sourced tissues carries with it a unique set of challenges. I think the authors can do a bit better with the way they present some of the data (more IHC images and better image quality) but overall I think they have put together a nice manuscript.

Thank you, we have improved the revised manuscript accordingly.

**Reviewer #2:**

The study provides promising data regarding the role of HLA-G in HAT. However, essential revisions are needed to improve methodological clarity, data presentation, and control group selection. Once these issues are addressed, the manuscript will be better positioned to make a significant contribution to the field.

Thank you, these aspects were clarified and improved in the revised manuscript.

**Reviewer #3:**

Overall, the paper by Lagrave et al is of great relevance, and of significant novelty in the context of better understanding of human African trypanosomiasis. They use a combination of immuno-histochemistry and ELISA to derive the conclusions shown. In general, the results are interesting. Major revision is requested, particularly in relation to Figures 1 and S1 (see above comments).

Thank you, we have improved the revised manuscript accordingly.
